# In Vitro Antagonistic Effect of Lactic Acid Bacteria Isolated from Fermented Beverage and Finfish on Pathogenic and Foodborne Pathogenic Microorganism in Ethiopia

**DOI:** 10.1155/2021/5370556

**Published:** 2021-10-15

**Authors:** Fitsum Dejene, Belayneh Regasa Dadi, Dagimawie Tadesse

**Affiliations:** Department of Medical Microbiology, Arba Minch University, Arba Minch, Ethiopia

## Abstract

**Background:**

Lactic acid bacteria from fermented foods and fish can antagonistically inhibit the growth of foodborne pathogenic organism in fermented food and they stimulate the immune response to protect the fish from certain kinds of infections. The aim of this study was to evaluate the in vitro antagonistic activities of lactic acid bacteria isolated from fermented beverage (Borde) and finfish on foodborne pathogenic microorganisms.

**Methods:**

Laboratory-based experimental study was conducted from May 1 to Sep 1, 2020. Total sample numbers were 60 samples of fermented beverage (Borde) and 20 of finfish which were collected from different households and Chamo Lake (Arba Minch, Ethiopia). Each sample was firstly homogenized and serial dilution was prepared and spread on MRS agar plates in order to isolate pure culture. Different biochemical tests were performed to identify isolated bacteria. Then, cell-free supernatant (CFS) was prepared from MRS culture and used in an antimicrobial assay that was performed by agar diffusion method. The effects of pH, temperature, and enzymes on antimicrobial activity were evaluated in the same test. Simultaneously, the effects of lactic acid bacteria on aflatoxin production and on the permeability of the membrane were also evaluated. Data were analyzed using one-way ANOVA and Tukey post hoc analysis was performed by SPSS 25 statistical software.

**Result:**

A total of 40 lactic acid bacteria were isolated; among them, 4 lactic acid bacteria, belonging to the genera *Enterococcus*, *Leuconostoc*, and *Weisellia* from fermented beverage and *Pediococcus* from fish, were screened for antimicrobial activity. The cell-free supernatant of those four isolates exhibited a significant (*p* < 0.05) antibacterial effect against tested pathogens and foodborne pathogenic bacteria. In addition, CFS showed antifungal and antiaflatoxigenic activities. The antimicrobial compounds synthesized by these isolates were sensitive to some proteolytic enzymes, and they were proved to be stable at high temperatures. It maintained/retained antimicrobial activity in a wide range of pH 2.0–10. Enterococcal CFS exhibited antibacterial activity against *S. aureus* on membrane permeability, as confirmed by the increase in absorbance value between 0.075 and 0.24 at OD_280-nm_ and between 0.68 and 1.2 at OD_260-nm_.

**Conclusion:**

Cell-free supernatant produced by isolated lactic acid bacteria showed antimicrobial activity against a wide range of Gram-positive and Gram-negative foodborne bacteria, suggesting its potential application as a natural antimicrobial agent in tackling the rising drug resistance against foodborne pathogens.

## 1. Background

All food should be safe and free of contamination at all points on its journey from the source to the consumers. However, food contamination is a serious public health problem in every country especially in Ethiopia, resulting in foodborne diseases that affect many people every year. For instance, in the USA, acute gastroenteritis affects 250 to 350 million people annually, and an estimated 22% to 30% of these cases are thought to be foodborne diseases with the main foods implicated including meat, poultry, eggs, seafood, and dairy products [1].

According to data from the Center for Disease Control and Prevention, it has been estimated that approximately one in four Americans may experience some form of foodborne illness each year. The bacterial pathogens that account for many of these cases include *Salmonella*, *Campylobacter jejuni*, *Escherichia coli* 0157:H7, *Listeria monocytogenes*, *Staphylococcus aureus,* and *Clostridium botulinum* [2–4]. In addition to this, some toxigenic *Aspergillus* strains such as *A. flavus* and *A. parasiticus* produce aflatoxin, an important carcinogenic mycotoxin with side effects such as malformations and immune suppression. The contamination of food and feed materials with aflatoxins, which have toxic, carcinogenic, and mutagenic activities, causes serious health problems and economic losses [5–8].

Until now, approaches to seeking improved food safety have relied on the search for more efficient chemical preservatives or on the application of more drastic physical treatments (e.g., high temperatures). Nevertheless, these types of solutions have many drawbacks: the proven toxicity of many commonly used chemical preservatives (e.g., nitrites), changes in organoleptic and nutritional properties of foods, and especially recent consumer trends in purchasing and consumption, with demands/requirements for safe but minimally processed products without additives [9].

In various ecological niches, microorganisms compete with each other for survival and through evolution form unique microbial communities. In some food ecosystems, lactic acid bacteria (LAB) constitute the dominant microflora. These organisms are able to produce antimicrobial compounds against competing flora, including foodborne spoilage and pathogenic bacteria. Under unfavorable environmental conditions, many species of LAB also produce exopolysaccharides (EPSs), which protect themselves against desiccation, bacteriophage, and protozoan attack [10–12].

A number of studies have shown that microorganisms from fermented foods can reach the gastrointestinal tract, this is likely to differ across products, and their presence in the gut appears to be transient [13]. Nonetheless, these microorganisms may still have the potential to exert a physiological benefit in the gut, through competition with pathogenic bacteria and the production of immune-regulatory and neurogenic fermentation by-products. Secondly, fermentation-derived metabolites may exert health benefits. For example, lactic acid bacteria (relevant to both dairy and nondairy fermented foods) generate bioactive peptides and polyamines with potential effects on cardiovascular, immune, and metabolic health [14, 15].

Most LAB are considered GRAS (generally recognized as safe) by the US Food and Drug Administration [16]. LAB is generally employed because they significantly contribute to the flavor, texture, and, in many cases, the nutritional value of the food products. LAB plays a defining role in the preservation and microbial safety of fermented foods, thus promoting the microbial stability of the final products of fermentation. Protection of foods is due to the production of organic acids, carbon dioxide, ethanol, hydrogen peroxide, and diacetyl, antifungal compounds such as fatty acids or phenyllactic acid, bacteriocins, and antibiotics such as reutericyclin [17].

A variety of fermented cereal beverages are produced in different parts of Ethiopia. These consist of different varieties of Tella, fermented beverage (Borde), Shamita, Korefe, and others. Fermented beverage (Borde) and Shamita are among the most important and popular fermented beverages consumed in the southern regions of Ethiopia. Fermented beverage (Borde) is produced by fermenting maize whereas barley is the major ingredient for Shamita production [18]. They are produced by an overnight fermentation of certain cereals predominantly by LAB. They are low-alcohol products and are consumed in large amounts as meal replacements [19].

Similarly, the fish gut microbiota plays an important role in GI tract development, digestive function, mucosal tolerance, stimulating the host immune response, and protection against infections. The GI tract in fish is one of the most important interfaces with the environment exposed to potential pathogens. The presence of LAB in GI tract of finfish helps them to antagonize certain pathogenic microorganisms [20]. The antimicrobial compounds from lactobacilli may inhibit bacteria as well as currently used antibiotics and chemical preservatives or applied drastic physical treatments so they may have clinical value in treatment of resistant microbial strains. Recently, many scientists have reported the presence of *Lactobacillus* in Borde and fish [16, 18, 19]. Due to a rise in drug resistance to commonly used antibiotics against foodborne pathogens globally [5–8], it is better to find alternatives to tackle drug resistance. One of the mechanisms is to use natural antimicrobial agents as an alternative to antibiotics. Therefore, the aim of this study was to evaluate the in vitro antagonistic activities of lactic acid bacteria isolated from fermented beverage (Borde) and finfish, on the growth of selected foodborne pathogenic microorganisms.

## 2. Methods

### 2.1. Study Design, Period, and Setting

Laboratory-based experimental study was conducted at Arba Minch University in Microbiology and Parasitology Laboratory, from May to September 2020. All experiments were performed in triplicate. Fermented beverage and fish were selected as a source for isolation of lactic acid bacteria (LAB) from different households in Arba Minch town and Chamo Lake (Arba Minch, Ethiopia). Arba Minch is located in Southern Nation, Nationalities and Peoples Region (SNNPR) regional zone and the administrative center of the Gamo Zone. It is located 504 km away from capital city Addis Ababa at 30°56′N of the equator and 37°44′E with a surface area of 2184 hectares with an average temperature of 30.6°C and annual rainfall of 575 mm [21]. Inclusion criteria include fermented food which was fresh and free from any contamination, fish that was found in adult stage, and fresh adult fish. Exclusion criteria include spoiled fermented food selected based on their texture and flavor which favors the growth of contamination, while larval stages of fish were not selected because they do not have enough amount of lactic acid bacteria in their gastrointestinal tract.

### 2.2. Sample Size Determination and Sampling Technique

The total sample number predicted to be taken in this experimental test was 60 samples of fermented beverage (Borde) (25 ml each) and 20 samples of finfish (at least 400 g each) that were randomly collected aseptically by using sterilized containers from different local households and fermented beverage (Borde) making microenterprises in Arba Minch, Ethiopia, and the finfish from Chamo Lake, Arba Minch, Ethiopia.

### 2.3. Data Collection and Laboratory Processing

#### 2.3.1. Sample Collection

Fermented beverage and fish were selected as a source for isolation of LAB. A food product and finfish which was subjected to study were 60 fermented beverage (Borde) samples and 20 samples of fish were collected from different households and Chamo Lake (Arba Minch, Ethiopia), respectively. The samples were kept in an icebox and transported to the laboratory within 3 hours; these samples were stored at −4°C until used for further analysis.

#### 2.3.2. Bacteria Used for Experimental Study

The standard strains used in this study were *Staphylococcus aureus* ATCC25923, *Klebsiella pneumonia* ATCC700603, *Pseudomonas aeruginosa* ATCC27853, *Escherichia coli* ATCC25922, and *Aspergillus flavus*. The strains were obtained from the collection center of Ethiopian Public Health Institute (EPHI) except *Aspergillus flavus* which was isolated and identified following growth on selective DzapexDox Agar (DDA) followed by morphological and microscopic characterization. All strains used in the experimental study were destroyed after the study was completed by autoclaving at a temperature of 121°C for 15 minutes using saturated steam under 15 psi pressures.

### 2.4. Isolation and Maintenance of Pure Cultures

Ten grams of each sample was homogenized with 90 ml of 0.85% (w/v) saline and serially diluted. One hundred microliters of the desired sample suspension was spread on MRS agar media. After plating the samples, the plates were incubated both aerobically and anaerobically at 37°C for 24 hrs. Colonies were selected randomly and purified by restreaking. Purified strains were stored as stock at −4°C till further use.

### 2.5. Phenotypic Identification and Biochemical Characterization of LAB

According to Kumar Colony morphology, cell morphology, arrangement, and Gram staining were done for phenotypic characterization. Colony shape (both elevation and margin), size, color, and consistency were observed. Other characteristics of the colonies such as smoothness (shiny glistening surface), roughness (dull, bumpy, granular, or matte surface), mucoidity (slimy or gummy appearance), and opacity (transparent, translucent, or opaque) were also noted. Gram staining was performed in order to categorize the isolates into Gram positive or Gram negative and rods, cocci, or coccobacilli. An endospore staining test was also performed to distinguish between spore and nonspore-forming bacteria. Endospores appeared bright green and vegetative cells brownish red to pink under microscope. Then, finally, the biochemical test was used to identify the isolated LAB species [22].

### 2.6. Preparation of Cell-Free Supernatants

Strains to be tested for antimicrobial activity were incubated in MRS broth for 48 h at 37°C. Bacterial cells were removed by centrifugation of the culture at 5000 × *g* for 20 min at 4°C. The pH values of supernatants were adjusted to pH 6.5–7.0 by the addition of 1 N NaOH. The supernatants were membrane filtered (Millipore, 0.22 *μ*m) and stored at 4°C until use [23].

### 2.7. Antimicrobial Assay

The antimicrobial activity of LAB was determined by a well-diffusion assay as described by Kojic et al. in 1991 [24]. In brief, the antimicrobial susceptibility was initially assayed by the agar well-diffusion method on the Mueller–Hinton agar (MHA). 100 *μ*l of each CFS was prepared. The selected pathogenic and foodborne pathogenic bacteria cell suspensions were adjusted to 0.5 McFarland turbidity standards to prepare 1 × 10^8^ bacteria/ml inoculums. Each bacterial suspension was inoculated on Mueller–Hinton agar plates, and the plates were then allowed to dry for 5 minutes. Wells were punctured in the medium then sealed with sterile molten agar. Cell-free supernatant (100 microliters) was added to the respective wells. MRS broth was used as a negative control. CFSs were neutralized to pH 6.5 or 7 to maintain that the inhibition was not due to lactic or other organic acids but by antimicrobial substances. After 48 h incubation at 37°C aerobically, the diameter of zones of inhibition (mm) was measured.

### 2.8. Effect of pH, Temperature, and Enzyme on CFS Antimicrobial Activity

Sensitivity to enzymes was checked by treatment with proteolytic enzymes: trypsin (BIO BASIC Canada INC) and pepsin and with lysozyme (BIO BASIC Canada INC); fifty microliters of the CFS was mixed with enzymes solutions to obtain final concentration and 1× reaction buffer. Incubation was held at 37°C for 1 h. After incubation, the enzymes were denatured by heating the samples at 80°C for 10 min, and the residual CFS activity was determined. The effect of pH on the CFS antimicrobial activity was determined by adjusting the pH of CFS using 1 N HCl, 1 N NaOH, and pH meter following that pH 2, pH 4, pH 6, pH 10, and pH 12 were used. After 2 h of incubation at room temperature, the samples were readjusted to pH 6.5 with sterile 1N HCl or 1 N NaOH, and the activity was determined. *Staphylococcus aureus* ATCC25923 was used as an indicator bacterium.

### 2.9. Effect of CFS on the Leakage of Cellular Metabolites

Bacterial culture of *S. aureus* ATCC25923 in 10 ml of nutrient broth (NB) at the exponential growth stage was transferred into several sterile centrifuge tubes and was centrifuged at 4,800 × *g* for 15 min. After the supernatant was discarded, the pellet was resuspended in 10 ml of NB, pH 7. The cell suspension was centrifugated twice and resuspended in 10 ml of NB. Bacterial suspensions of the *S. aureus* ATCC25923 from all centrifuge tubes were pooled, and OD_540_ values and viable counts of bacteria were determined. Then 10 ml aliquots were dispensed into each of six sterile flasks (50 ml). 1 ml of CFSs was added to cells and control (without CFS) was prepared. At 1-, 2-, and 3-hour time intervals of treatment, cells were centrifuged at 3,500 × *g*, the suspension was filtered through 0.22 *μ*m sterile filters, and the filtrate was used for determination of nucleic acids at A260 nm and proteins at A280 nm. The differences of absorption value at A260 and A280 nm between controls and test groups were used to estimate/quantify the release of metabolites. The experiments were done in triplicate [25].

### 2.10. Effect of Lactic Acid Bacteria on Growth Rate and Aflatoxin Production of *A. flavus*

The spore suspension was prepared from pure cultures of *Aspergillus flavus* (21 days old) grown on the Czapexdox medium. The plates were flooded with 0.05% Tween 80 and brushed thoroughly for 1-2 min with a sterilized slide. The suspension was filtered through three layers of sterile cheesecloth to remove the mycelia residues. The number of spores/ml was counted in the collected spore suspension by using a Neubauer-improved haemacytometer and was adjusted to 20 × 10^4^ spores/ml in Czapexdox Medium [26].

The effect of lactobacilli on aflatoxin production by *Aspergillus flavus* was tested by inoculating 2 mL of bacterial CFS in 50 ml yeast extract sucrose broth (YESB) supplemented with a standard amount of fungal spores (20 × 10^4^ spores/ml), followed by incubation at 28°C for 20 days. YESB media supplemented with known fungal spores and YESB media without any inoculation were incubated as positive and negative controls, respectively. After incubation, medium containing CFS and fungus was filtered through sterile Whatman filter paper no. 1, fungal mycelial mass was weighed after drying in an oven at 70°C, and aflatoxin B1 quantity in filtrate was measured by Enzyme-Linked Immune Sorbent Assay (ELISA) and compared with controls [27]. Aflatoxin was detected by ELISA and quantified using the calibration curve.

(1) $$\begin{array}{c} \text{Percentage of Absorbance}\left(\%\right)=\frac{B}{B0}*100\%, \end{array}$$

where *B* is the absorbance of standard or sample, *B*0 is the absorbance of zero standards, and % age reduction = 1 − ((concentration of AFB1 in treatment)/(concentration of AFB1 in control)).

### 2.11. AFB1 Extraction for ELISA Analysis

Aflatoxin B1 was extracted by adding 25 ml chloroform to each culture flask which was then shaken for 15 minutes on a rotary shaker model no. RSE056 (150–160 rpm). After phase separation by rotary evaporator S/N 200095320, the chloroform layer was removed and the extraction was repeated with additional 25 ml chloroform. Combined extracts were dehydrated over granular anhydrous sodium sulfate and evaporated to dryness at 60^◌^C in a water bath. Residues were dissolved in 1 ml of chloroform for analysis. Aflatoxin B1 concentration was determined by ELISA [26].

### 2.12. Data Quality Assurance

Data quality control was ensured from data collection up to final laboratory identification by following the prepared standard operating procedure (SOP). The performance of the prepared media was checked by inoculating control strains like *Staphylococcus aureus* ATCC25923 and *Escherichia coli* ATCC25922, which was obtained from Ethiopian Public Health Institute (EPHI). Culture media was prepared according to manufactures instruction, and the sterility was checked by incubating 5% of prepared media at 35–37°C overnight and observing bacterial growth. Those batches of the media that show the growth were discarded and reprepared. Data quality control for the experimental unit is Positive and Negative Control Unit.

### 2.13. Statistical Analysis

All experiments were performed in triplicate. Data were analyzed within one-way ANOVA and statistical analysis was performed using post hoc Tukey test by SPSS 25 (IBM Statistics, Armonk, NY) statistical software. *p* ≤ 0.05 was considered as the statistical significance level.

## 3. Results

### 3.1. Morphological Identification

The identification was performed for four isolates, which is according to the morphological and microscopic characteristics. The macroscopic appearance of all colonies was examined for morphological characteristics. The size, shape, color, and texture of colonies were recorded. The macromorphological characteristics of different isolates studied on MRS agar plates are summarized in Table 1.

Through Gram staining and microscopy, the isolates were differentiated into Gram-positive cocci or coccobacilli. Out of 4 isolates, all were found to be Gram-positive. Out of all samples, 4 different bacteria were isolated of which micromorphologically 2 were cocci and 2 were coccobacilli (Table 2).

### 3.2. Biochemical Characterization

After morphological studies, various biochemical tests were performed with isolated bacteria in order to identify isolates on the basis of their biochemical characteristics. All 4 isolates were catalase-negative and nonmotile and all were fermentative. Some of the isolates could grow at 15°C and some of the strains were able to grow at 45°C (Table 3).

Further biochemical analysis was done through which 8 carbohydrates utilization tests were performed. By studying the results of this test, different isolates could be tentatively characterized on the basis of their biochemical nature (Table 4). According to the biochemical tests, the 4 isolates could be tentatively categorized into *Enterococcus* sp., *Pediococcus* sp., *Weissella* sp., and *Leuconostoc* sp., and *Enterococcus*, *Leuconostoc*, and *Weissella* were isolated from fermented beverage (Borde), while *Pediococcus* was isolated from finfish.

### 3.3. Antibacterial Potential of LABCFS on Pathogenic Organism

The antibacterial activity of CFS of all isolates against the tested foodborne pathogenic bacteria was confirmed by the presence or absence of inhibition zones around well on the agar plates. As presented in Table 5, CFS of some isolates exhibited potent inhibitory effects against all the tested foodborne pathogenic bacteria. In this assay, most isolates exerted consistent antibacterial effects against both Gram-positive and Gram-negative bacteria, with a zone of inhibition diameters ranging from 7 to 11 mm for neutralized CFS, while it was 13 to 20 mm for nonneutralized CFS. MRS medium, used as a negative control, had no inhibitory effect (Table 5).

Generally, when inhibition zones produced by isolates belonging to each LAB genus were compared, there was a statistically significant difference between groups as demonstrated by one-way ANOVA [*F* (4, 15) = 7.238, *p*=0.002]. A Tukey post hoc test showed that there was no statistically significant difference between the groups, However, there is a statistically significant difference in comparison with the negative control (*p* < 0.05) except the group that is treated by *Weissella* and *Leuconostoc*. Among the test strains, the most sensitive were *S. aureus* ATCC25923 for *Enterococcus*, *Pediococcus,* and *Weissella,* while *K. pneumonia* ATCC700603 was more sensitive to *Leuconostoc* isolate. *E. coli* ATCC25922 was the least sensitive in all cases.

### 3.4. Inhibitory Activity of CFS following Heat Treatments

The CFS produced by the four selected LAB isolates were heat-treated at 60 and 100°C for 10 min. The antimicrobial compound in CFSs was thermally stable under these heat conditions above as their inhibitory effects against *S. aureus* were observed (Table 6). Nevertheless, CFS lost activity after temperature treatment at 121°C for 10 min displaying no inhibition zones compared to the control.

### 3.5. Inhibitory Activity of CFS following Enzyme Treatments

The sensitivity of the CFS to trypsin, pepsin, and lysozyme was determined in controlled and reproducible conditions as shown in Table 7. The inhibitory substance was fully inactivated by proteolytic enzymes, while the lysozyme enzyme had no effect on the antagonistic activity.

### 3.6. Inhibitory Activity of CFS at Different pH Condition

The exposure of CFS to different pH values showed that all antimicrobial substances remained fully active in the pH range from 2–8 and also at pH 10. Reduced activities of antimicrobial substances from CFS of all isolates were found after treatment at pH 12.0 (Table 8).

### 3.7. Effect of CFS on the Release of Intracellular Component Materials

The ODs of culture filtrates of *S. aureus* ATCC25923 cells exposed to the CFS of *Enterococcus* at 260 nm and 280 nm revealed a significant time-dependent increase in the release of 260 nm and 280 nm absorbing materials. However, no changes in the OD of control cells of the tested pathogen were observed.

The absorption of the material cell at 260 and 280 nm in spectrophotometer UV from filtrated samples and control suspension were significantly different. The absorption value indicates that there is leakage of intracellular macromolecules. Molecules that absorb at 260 nm are nucleic acids and at 280 nm are proteins. Significant increases in the absorption at 260 nm and 280 nm occurred after treatment with CFS.

### 3.8. Antifungal Potential of LABCFS on *Aspergillus flavus*

All the studied LAB strains exhibited various degrees of growth inhibition against *Aspergillus* species. All tested LAB strains showed antifungal activity against *A. flavus.* The indicator strain *A. flavus* was the most sensitive to *Enterococcus* isolate that was followed by *Pediococcus*, *Leuconostoc,* and *Weissella*.

Each isolate significantly reduced the growth rate of *A. flavus* in comparison with the positive control as demonstrated by one-way ANOVA (*F* (4, 10) = 113.386, *p* ≤ 0.001). However, there was no significant difference between the antifungal activities of these four isolates (Table 9).

### 3.9. Antiaflatoxigenic Effect of LABCFSs on *Aspergillus flavus* AFB1 Production

The production of Aflatoxin B1 was greatly affected by the presence of all the investigated LAB species. Data presented in Table 10 indicate that the inhibitory effect on Aflatoxin B1 production by *A. flavus* ranged from 98.1% in the presence of *Leuconostoc* to 99.8% in the presence of *Enterococcus* after 15 days of incubation. Based on the differences in antifungal activity by LAB cultures and the controls, *Enterococcus* isolate was the most antifungal (79.7% growth reduction) and antiaflatoxigenic (99.8% reduction of AFB_1_ production) to the test strains, while *Weissella* was the weakest antifungal and antiaflatoxigenic isolate.

All isolates significantly reduced the production of AFB_1_ (Aflatoxin B1) in comparison with the positive control as demonstrated by one-way ANOVA (*F* (4, 10) = 533.307, *p* ≤ 0.001). However, there was no significant difference between the antiaflatoxigenic activities of these four isolates (Table 10).

## 4. Discussion

In our experimental study, 4 lactic acid bacteria isolated from finfish and fermented beverage (Borde) were identified based on its micromorphological, biochemical, and fermentation abilities test result. In this study, 4 isolates of LAB were screened for its antibacterial activities against the pathogenic and foodborne pathogenic organisms. As confirmed by inhibitory zones in the agar well-diffusion assay *Pediococcus*, *Enterococcus*, *Leuconostoc*, and *Weissella* were used to investigate the antagonistic activity. As reported previously, several LAB have shown potential antibacterial effects against a number of pathogenic and foodborne pathogenic organisms [28–30]. In this assay, 4 strains of pathogenic and foodborne pathogenic bacteria (*S. aureus* ATCC25923, *E. coli* ATCC25922, *K. pneumonia* ATCC700603, *P. aeruginosa* ATCC27853), and *A. flavus* were used to evaluate the antagonistic effects of CFS of those 4 isolates.

The antibacterial activity of the four LAB strains confined from fermented beverage (Borde) and finfish has been examined primarily in vitro, and studies have especially centered on the inhibitory activity against the growth of Gram-negative and Gram-positive pathogens. An antibacterial substance of CFS produced by *Enterococcus*, *Pediococcus*, *Leuconostoc*, and *Weissella* strains exhibited inhibitory activity against all four bacterial strains *S. aureus*, *E. coli*, *K. pneumonia*, and *P. aeruginosa*. A recent review has similarly demonstrated that isolated species of *Lactobacillus* exert antagonistic impact in vitro against pathogenic and foodborne pathogenic organisms by creating antibacterial metabolites, most of which remain to be recognized [31, 32]. There is expanding prove that the antibacterial action of LAB includes various mechanisms of action, including the generation of lactic acid and antimicrobial substances like bacteriocins and nonbacteriocin molecules [33–35].

The CFS antagonistic activity of isolated bacteria can be influenced by a few proteolytic enzymes driving to loss in their antagonistic action. In this study, *S. aureus* was utilized as an indicator organism and the CFS of LABs had diverse inhibitory impact taking after treatment with a proteolytic enzyme. A comparable character was observed by *Lactobacillus plantarum* bacteria. They found that proteolytic enzymes like pepsin and trypsin treatment hindered the antagonistic activity of test strain [36]. Scatassa and his colleague also reported that the strain of *Lactobacillus* exhibited similar antibacterial activity, inhibiting *L. monocytogenes* ATCC 7644 and eight *L. monocytogenes* of food origin and the proteolytic enzyme treatment of cell-free supernatant of *Lactobacillus* eliminated all inhibitory activity, confirming that the toxins were proteinaceous in nature [37].

The antagonistic activity of CFS illustrated by the four isolates was too pH-dependent. The foremost antagonistic activity was displayed in the acidic pH range of 2 to 5, whereas loss of antagonistic was observed in alkaline pH condition (pH > 10). The same result was reported by Pehrson et al. in which lactic acid bacteria, specifically *L. acidophilus* ATCC 4356 obtained from the research center, appeared to have the most elevated antagonistic activity and stability at pH 2 and 4 [29].

The stability of the antagonistic compound of CFS was also analyzed at diverse temperatures. The CFS produced by the four isolates was considered as moderately stable since no reduction in activity after heating at 60°C and 100°C for 10 min was observed, whereas it lost its activity at above 100°C. The same result was also reported showing loss of activity after heat treatment at 121°C for 15 min [36]. Furthermore, Nowroozi and his colleague also reported that lactic acid bacteria isolated from sausage had antibacterial activity and the antibacterial activity was stable at 100°C for 10 minutes and at 56°C for 30 minutes but actively was lost after autoclaving [38].

In this study, it was demonstrated that CFS of *Enterococcus* species had exceptional impacts on the discharge of 260 nm and 280 nm materials like nucleic acids (DNA; RNA) and proteins from the cells of tested *S. aureus* ATCC25923 bacteria, which affirmed its potential as a strong antibacterial agent. According to Bajpai et al., marked discharge of 260 nm and 280 nm materials from CFS treated cells of pathogenic bacteria was supported by the observation of loss of cell membrane structural integrity, which would lead to the loss of basic cell metabolite [39]. These results recommend that the leakage of 260 nm and 280 nm absorbing materials from *S. aureus* might give sensitive indicator membrane damage and loss of membrane integrity. Comparable results on the inhibitory impact of CFSs on nucleic acid and protein leakage from bacterial pathogens have been previously reported [40]. Numerous antimicrobial compounds that act on the bacterial cytoplasmic layer initiate the loss of 260 nm absorbing materials (nucleic acid) and proteins as 280 nm absorbing material [36].

In the investigation with respect to the antifungal and antiaflatoxigenic activity of LAB, *Enterococcus* and *Pediococcus* caused a basic reduction within the mycelial development of *A. flavus.* Besides, LAB had shown inhibitory impacts on the production of Aflatoxin B1 from 98.8 to 99.8% in comparison with the control. The result of this study is in agreement with the study conducted within the College of Nebraska-Lincoln, USA, in which three of LAB of *L. plantarum*, *L. rhamnosus*, and *L. paracasei* hindered the growth of mold and mycotoxin producing *A. flavus* and *A. parasiticus* [26]. The same study was conducted by Rafaat in which the antifungal action by lactic acid bacteria hindered both the growth and aflatoxin production of *A. flavus* [32, 41]. Similarly, Erick and his colleague investigated that thirteen out of the 18 tested lactic acid bacteria showed antifungal activity. Almost all lactic acid bacteria supernatants tested showed antifungal activity at a certain growth phase, probably due to the acidifying of the supernatants [41]. Furthermore, Aiko and Mehta similarly reported that few strains of lactic acid bacteria showed an ability to remove aflatoxins B1 and M1 by binding noncovalently indicating that binding and not metabolism is the mechanism by which the toxins are removed [42]. The inhibition of fungal development and aflatoxin production by these bacteria likely is due to different factors such as nutrient competition, secondary metabolites, pH, or their combinations [34, 40].

Overall our findings indicate that LAB can be used to inhibit the growth of pathogenic and/or potentially pathogenic microorganisms and can be used as an alternative antimicrobial agent as there is a growing concern of antibiotic resistance to drugs used for foodborne pathogens. In addition, it is safe to use LAB to preservative foods in industries rather than using a chemical preservative.

### 4.1. Limitations of the Study

HPLC analysis and purification of antagonistic metabolite were not performed due to the fact that the machine currently is not giving any service because of the COVID-19 pandemic. Species identification was not performed due to the lack of RAPD-PCR primer and API 50CH *Lactobacillus* identification KIT. Effect of CFS on cell morphology and intracellular organization was not performed due to the fact that the SEM machine was not operational during the research period.

## 5. Conclusions

Cell-free supernatant produced by the four isolates of LAB exhibited antimicrobial activity against a wide range of Gram-positive and Gram-negative foodborne bacteria, suggesting its potential research and application as a natural food antimicrobial agent. This antibacterial and antifungal LAB can be used in the food industry instead of chemical preservatives to produce organic foods. Furthermore, the excellent properties of LAB may preserve the nutritional value of foods and delay spoilage. From a health perspective, the conventional antimicrobial agents usually provide effective antibiotic therapy for bacterial infections but today many of the antimicrobial agents fail to respond to treatment and resulting in prolonged illness and greater risk of death. So using antimicrobial compounds from LAB may inhibit bacteria than the presently used antibiotics that mean it may have clinical value in treating certain microbial infection.

## Figures and Tables

**Table 1:** Morphological characteristics of colonies of different bacterial isolates.

Isolates	Form	Elevation	Margin	Surface	Opacity	Chromogenesis
*Leuconostoc*	Circular	Convex	Entire	Smooth	Opaque	Off white
*Enterococcus*	Circular	Flat	Entire	Smooth	Opaque	White
*Weissella*	Circular	Convex	Entire	Smooth	Opaque	Yellowish white
*Pediococcus*	Circular	Convex	Entire	Smooth	Opaque	Yellowish white

**Table 2:** Micromorphology of bacteria isolated from Borde and finfish.

Isolates	Naming (arrangement)	Types of colony
*Leuconostoc*	Cluster	Coccobacilli
*Enterococcus*	Diplococcic or short streptococci	Cocci
*Weissella*	Tetragenococcus	Cocci
*Pediococcus*	Cluster	Coccobacilli

**Table 3:** Summary of different micromorphological/biochemical tests of isolates.

Isolates	Gram staining	Catalase test	Citrate utilization test	Hydrogen sulfide production	Motility test	Indole test	Methyl red test	Growth at 15°C	Growth at 45°C
*Leuconostoc*	+	−	−	−	Nonmotile	−	+	+	−
*Enterococcus*	+	−	−	−	Nonmotile	−	+	+	+
*Weissella*	+	−	−	−	Nonmotile	−	+	+	−
*Pediococcus*	+	−	−	−	Nonmotile	−	+	+	−

**Table 4:** Summary of carbohydrate utilization test.

Fermentation test	Lactose	Glucose	Sorbitol	Maltose	Fructose	Sucrose	Starch	Mannitol
*Leuconostoc*	+	+	−	+	+	+	+	+
*Enterococcus*	+	−	+	+	+	+	−	+
*Weissella*	−	+	+	−	+	−	−	+
*Pediococcus*	+	+	+	+	+	+	−	+

**Table 5:** Inhibition of various indicator organisms by CFS produced by lactic acid bacteria.

Isolates	*E. coli* ATCC25922	*S. aureus* ATCC25923	*K. pneumonia* ATCC700603	*P. aeruginosa* ATCC27853
*Enterococcus* NCFS	16 mm	19 mm	18 mm	18 mm
*Enterococcus* NNCFS	7 mm	11 mm	9 mm	10 mm
*Pediococcus* NNCFS	18.4 mm	17 mm	18 mm	17 mm
*Pediococcus* NCFS	8 mm	12 mm	11 mm	10 mm
*Leuconostoc* NNCFS	17 mm	15 mm	17 mm	8 mm
*Leuconostoc* NCFS	8 mm	7 mm	9 mm	ND
*Weissella* NNCFS	15 mm	17 mm	9 mm	10 mm
*Weissella* NCFS	8 mm	9 mm	ND	ND
Negative control (MRS) broth	ND	ND	ND	ND

*Note*. ND: not detected; NNCFS: nonneutralized CFS; NCFS: neutralized CFS.

**Table 6:** Thermal stability of CFS produced by the selected LAB following various heat treatments (*S. aureus* ATCC25923 was used as an indicator bacterium).

Isolates	60°C, 10 min	100°C, 10 min	121°C, 10 min
*Enterococcus* NCFS	++	+	−−
*Pediococcus* NCFS	++	+	−−
*Leuconostoc* NCFS	++	+	−−
*Weissella* NCFS	++	+	−−
MRS + enzyme	−−	−−	−−

*Note*. ++ = inhibition zone (7–10 mm); + = inhibition zone (<5 mm); −− = no inhibition zone.

**Table 7:** Inhibitory activity of CFS following enzyme treatments (*S. aureus* ATCC25923 was used as an indicator bacterium).

Isolate	Trypsin	Pepsin	Lysozyme
*Enterococcus* NCFS	−	−	++
*Pediococcus* NCFS	−	−	++
*Leuconostoc* NCFS	−	−	++
*Weissella* NCFS	−	−	++
MRS + enzyme	−	−	−

*Note*. ++ = inhibition zone (7–10 mm); + = inhibition zone (<5 mm); − = no inhibition zone.

**Table 8:** Inhibitory activity of CFS following different pH treatments (*S. aureus* ATCC25923 was used as an indicator bacterium).

Isolates	pH 2	pH 4	pH 10	pH 12
*Enterococcus* NCFS	++	++	++	+
*Pediococcus* NCFS	++	++	++	+
*Leuconostoc* NCFS	++	++	++	+
*Weissella* NCFS	++	++	++	+
MRS broth	+	+	+	+

*Note*.++ = inhibition zone (20–13 mm); + = inhibition zone (<13 mm).

**Table 9:** Mycelial mass production by *A. flavus* in the presence of LAB.

Species	Mycelial mass (gm)	Percent of mycelial mass reduction (%)
*A. flavus* + *Enterococcus* CFS	0.16	79.7
*A. flavus* + *Pediococcus* CFS	0.20	74.7
*A. flavus* + *Leuconostoc* CFS	0.23	70.9
*A. flavus* + *Weisella* CFS	0.27	65.8
*A. flavus*	0.79	—

**Table 10:** Effect of LABCFS on the concentration of AFB_1_ produced by toxigenic *A. flavus*.

Species	AFB_1_ (ppm)	AFB_1_ percent of reduction (%)
*A. flavus* + *Enterococcus* CFS	46.3	99.8
*A. flavus* + *Pediococcus* CFS	135	99.3
*A. flavus* + *Leuconostoc* CFS	403.3	98.1
*A. flavus* + *Weisella* CFS	252	98.8
*A. flavus*	20690	—

## Data Availability

The datasets generated and/or analyzed during the current study are not publicly available due to ethical and confidentiality reasons but are available from the corresponding author upon reasonable request under the ethics committee's approval.

## Abbreviations

AFB1: Aflatoxin B1
ANOVA: Analysis of variance
CFS: Cell-free supernatants
DDA: DzapexDox agar
ELISA: Enzyme-linked immunosorbent assay
EPSs: Exopolysaccharides
GRAS: Generally recognized as safe
LAB: Lactic acid bacteria
MDR: Multidrug resistance
MHA: Muller–Hinton agar
MRS: De Man, Rogosa, and Sharpe
NaOH: Sodium hydroxide
OD: Optical density
SOP: Standard operating procedure
SPSS: Statistical Package for Social Sciences
YESB: Yeast extract sucrose broth.

## Additional Points

The standard strains used for this experimental study were *Staphylococcus aureus* ATCC25923, *Klebsiella pneumonia ATCC700603*, *Pseudomonas aeruginosa* ATCC27853, *Escherichia coli* ATCC25922, and *Aspergillus flavus*. The strains were obtained from the collection center of Ethiopian Public Health Institute (EPHI) except *Aspergillus flavus* which was isolated and identified following growth on selective DzapexDox Agar (DDA) followed by morphological and microscopic characteristics. These strains used for experimental study were destroyed after the study was completed by autoclaving at a temperature of 121°C for 15 minutes using saturated steam under 15 psi pressure.
